# Impacts of sleep on the characteristics of dental biofilm

**DOI:** 10.1038/s41598-020-80541-5

**Published:** 2021-01-08

**Authors:** Maki Sotozono, Nanako Kuriki, Yoko Asahi, Yuichiro Noiri, Mikako Hayashi, Daisuke Motooka, Shota Nakamura, Hiroyuki Machi, Tetsuya Iida, Shigeyuki Ebisu

**Affiliations:** 1 Department of Restorative Dentistry and Endodontology, Osaka University Graduate School of Dentistry, 1-8 Yamadaoka, Suita, Osaka 565-0871 Japan; 2 Division of Cariology, Operative Dentistry and Endodontics, Department of Oral Health Science, Niigata University Graduate School of Medical and Dental Sciences, Niigata, Japan; 3 Research Institute for Microbial Diseases, Osaka University, Osaka, Japan; 4 Osaka University Dental Technology Institute, Osaka, Japan

**Keywords:** Microbiology, Health care

## Abstract

Dental biofilm present on the tooth surface is associated with oral diseases, such as dental caries and periodontal disease. Because bacterial numbers rapidly increase in saliva during sleep, oral care before sleeping is recommended for the prevention of chronic oral diseases. However, temporal circadian changes in the quantity and quality of dental biofilms are poorly understood. This study aimed to investigate the impacts of sleeping on dental biofilm amounts and compositions by using an in situ model. The use of this in situ model enabled us to investigate dental biofilm formed in the oral cavity and to perform a quantitative analysis. Subjects began wearing oral splints in the morning or before sleeping, and biofilm samples were collected at 8, 16, and 24 h after the subjects began wearing oral splints; these samples were then used in various experiments. No significant changes in the numbers of biofilm-forming bacteria were caused by sleep. However, the relative abundances of genera related to periodontitis (i.e., *Fusobacterium* and *Prevotella*) increased after awakening. In conclusion, the numbers of biofilm-forming bacteria were not affected by sleep, and the abundances of obligate anaerobes increased after sleep. This research may aid in defining efficacious preventive oral care.

## Introduction

The human body is inhabited by an enormous number of bacteria, which form a structure known as a biofilm in habitats such as the skin, airways, gut, vagina, and oral cavity^1^. More than 700 species of bacteria inhabit the human oral cavity^2,3^; they form biofilms on various locations, including the tooth surface, hard and soft palate, buccal mucosa, and gingiva. The Human Microbiome Project (HMP, 2007–2017) was established to investigate the characteristics of the human microbiome and its relationship to disease. In this project, the microbiomes of various habitats in the human body were comprehensively investigated by performing a 16S rRNA sequence analysis^1^. The oral cavity is reportedly one of the most microbiologically diverse sites within the human body^4^. Most bacteria detected in the oral cavity belong to one of the following five phyla: Firmicutes, Proteobacteria, Actinobacteria, Bacteroidetes, and Fusobacteria^5,6^. The diversity of the salivary and dental plaque microbiome differs widely among individuals. Because the oral cavity acts as the primary entrance to the human digestive tract, it tends to be influenced by host behaviors, such as toothbrushing and mouth washing^7^. In addition, the microbiome in the oral cavity is also affected by many other factors, including saliva pH, enzymes, host immunity, and antibacterial agents^8^.

Dental biofilm that forms on the tooth surface is thought to be associated with oral diseases, such as dental caries and periodontal diseases^9,10^, which are chronic infectious diseases found in many patients worldwide^11^. An imbalance of the microbiome (dysbiosis) contributes to some diseases, e.g. intestinal diseases, diabetes mellitus, and metabolic disease^12–14^. Recently, the dysbiosis of microbial communities in the oral cavity was found to be associated with oral infections^15,16^. For example, dental caries were found to result from a dysbiosis with acidogenic and aciduric bacteria, whereas periodontitis is triggered by dysbiosis with proteolytic, obligate anaerobic, and alkaliphilic bacteria^17^. Therefore, the prevention of these oral diseases requires monitoring and control of the microbial communities within dental biofilm. Biofilms, including dental biofilm, are composed of bacterial cells and exopolysaccharide (EPS)^8^. Importantly, biofilm-forming bacteria exhibit gene expression patterns that differ from those of planktonic bacteria^18,19^; moreover, a subset of bacteria in biofilms behave as persister cells, characterized by slow growth^20,21^. These mechanisms enable biofilms to tolerate exposure to typical antibiotics^22–24^. Therefore, the most effective mechanical approach is daily toothbrushing to remove the dental biofilm, which prevents dental caries, periodontitis, and other oral diseases.

Salivary flow is reportedly lower during sleep than during the daytime^25^; additionally, because the number of bacteria in saliva increases rapidly at night, it is highest upon awakening^26^. Accordingly, it is generally recommended that oral care (including toothbrushing) should be performed before sleeping. Patients and clinicians generally agree on the need for oral care before sleep. However, this practice is recommended solely based on the number of bacteria in saliva, and it does not consider the role of dental biofilm in the onset of oral disease. The salivary microbiome is reportedly associated with circadian oscillation^27^; however, there remains a lack of information regarding the relationship between dental biofilm and the circadian rhythm, as well as regarding the roles of changes in the abilities of dental biofilms to cause oral disease between waking and sleeping hours. Unfortunately, the effects of sleep on the characteristics of dental biofilm have not been sufficiently investigated owing to the difficulty of such experiments.

The concept of dental biofilm was originally reported by Costerton et al.^28^; various experiments have since been performed to investigate and control biofilm formation^29,30^. Moreover, many kinds of biofilm models have been developed to investigate human dental biofilm, including in vitro static models and flow cell models^31–34^. However, these in vitro models can neither simulate the environment of the oral cavity nor reflect the influence of host factors. Although these problems could potentially be solved by collecting dental biofilm directly from the tooth surface, the structure of collected biofilm would be disturbed by the instruments used for their collection. In previous studies, the growth rate and growth patterns of dental biofilm formed during the daytime and nighttime were recorded by taking photos, which subsequently were used to calculate the area covered by dental biofilm^35,36^. However, these experiments did not provide an accurate assessment of the amount of dental biofilm.

In our previous work, we developed an in situ dental biofilm model^37^ that enables the formation of experimental dental biofilm on hydroxyapatite (HA) disks in the oral cavity, which facilitates quantitative analysis. The use of this in situ dental biofilm model enables the collection of samples of dental biofilm formed in oral cavity without disturbing the biofilm structure and investigations of the amount of biofilm per unit area. We previously studied the temporal dynamics of experimental dental biofilm by using this in situ dental biofilm model for 96 h. We observed that, 48 h after the biofilm began to form, the bacterial number and biofilm thickness significantly increased; concurrently, the composition of the microbiota changed. Using a 16S rRNA sequence analysis, we found that mature dental biofilm (after 48 h of growth) had a high abundance of anaerobic bacteria, such as *Fusobacterium*, *Prevotella*, and *Porphyromonas* genera.

The purpose of the present study was to use our in situ model to investigate the impact of sleeping on the bacterial microbiota within dental biofilm. The findings of this study will contribute to improvements in preventive oral care based on an improved understanding of dental biofilms.

## Methods

### Selection of study subjects

Ten healthy volunteers (seven men and three women, 27–32 years old) were recruited from among the students and staff of Osaka University Graduate School of Dentistry. Healthy subjects were defined as previously reported^38^. No clinical signs of caries, gingivitis, or periodontitis were detected, and no systemic disease was observed in any of the subjects. The total number of decayed, missing, or filled teeth (DMF) of each participant was recorded as an index of dental caries, and the Community Periodontal Index (CPI) of each participant was recorded as an index of periodontal disease. A summary of the subject characteristics is shown in Table 1. The subjects were asked to avoid using antibiotics for 3 months before beginning this study.

**Table 1:** Subject characteristics.

Subject number	Sex	Age (years)	DMF	CPI
1	F	30	14	0
2	M	30	0	0
3	F	28	12	0
4	F	30	2	0
5	M	32	3	0
6	M	27	1	0
7	M	27	4	0
8	M	32	7	0
9	M	31	0	0
10	M	31	8	0

The subject characteristics are shown. F, female; M, male. A quantification of the experience of dental caries as the total number of teeth that are decayed, missing, or filled (DMF) was used as an index of dental caries. The Community Periodontal Index (CPI) was used as an index of periodontal disease. No clinical signs of caries, gingivitis, or periodontitis were detected, and no systemic disease was observed, in any of the subjects.

### Ethics declarations

Written informed consent was obtained from all subjects. The study design was reviewed and approved by the Ethics Committee of the Osaka University Graduate School of Dentistry (H29-E17). The experiments were performed in accordance with ethics guidelines concerning medical science studies of humans.

### Biofilm formation

**Figure 1:**
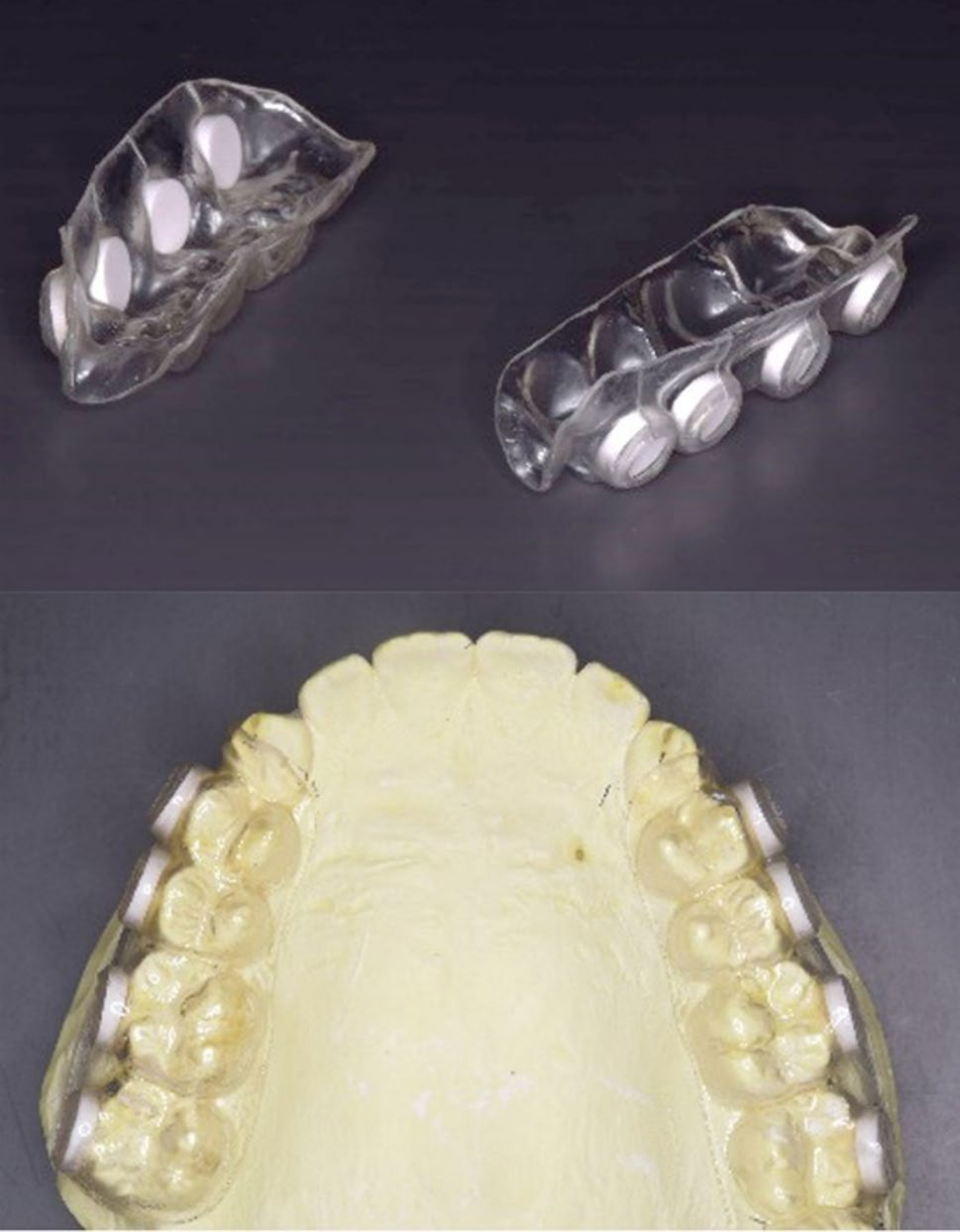
In situ dental biofilm model. Schematic of the in situ dental biofilm model. Oral appliances with HA disks inserted into the buccal sides were used to observe the formation of experimental biofilms.

The in situ dental biofilm model that we developed previously^37^ was modified and used to form biofilms. Briefly, individual upper jaw splints as in situ oral devices were vacuum-formed with 1.5-mm-thick thermoplastic resin sheets (Erkodule, Erkodent, Pfalzgrafenweiler, Germany) and divided into left and right pieces. HA disks (Olympus Terumo Biomaterials, Tokyo, Japan) simulating the enamel surface of teeth were inserted into the buccal side of the appliances (Fig. 1). All subjects brushed their teeth, without using toothpaste or mouthwash, before they inserted the oral appliance.

**Figure 2:**
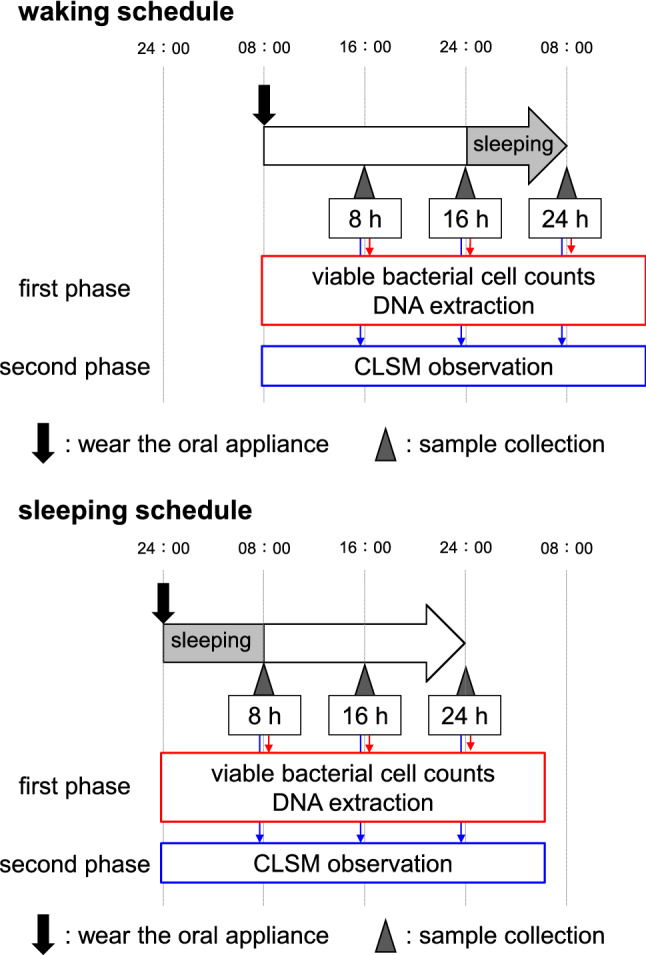
Schedule and sample collection timings. Schematic of the schedule and sample collection timings. Subjects began wearing the oral appliances (indicated by a black arrow) at 8:00 for the waking schedule and at 24:00 for the sleeping schedule. Biofilm samples were collected after wearing the oral appliances for 8 h, 16 h, or 24 h, as indicated by arrowheads. In both the waking and sleeping schedules, the subjects wore the oral appliances for 24 h and slept from 24:00 to 8:00. In the first phase, disks were collected for use in viable bacterial cell counts and DNA extraction (for real-time PCR and 16S rRNA sequence analysis). In the second phase, disks were collected at each sample collection timepoint for confocal laser scanning microscopy (CLSM) observations.

The experimental schedule is shown in Fig. 2. All ten subjects participated in both experimental schedules (waking and sleeping). A rest period of least 2 weeks was implemented between participation in the waking and sleeping schedules. Subjects began to wear the oral appliance in the morning for the waking schedule and directly before sleeping for the sleeping schedule. For both schedules, subjects wore the oral appliance continuously for 24 h, except for during meals and toothbrushing. When the subjects removed their oral appliances for eating and toothbrushing, they placed the appliances in cases with wet paper to avoid desiccation of the biofilms on the HA disks. After meals, the subjects brushed their teeth before resuming appliance wear, and they recorded the time of the meal in experimental time. For both schedules, the subjects slept for 8 h (from 24:00 to 8:00) and recorded their wake-up time and bedtime. Biofilm samples were collected at 8, 16, and 24 h after insertion of the oral appliances. Because eight HA disks were inserted in the appliance, and the number of biofilm samples formed at a single time was limited, each schedule was repeated twice (first and second phases, respectively), such that each participant wore the appliance for 4 days in total. The subjects participated in the different schedules in a randomly assigned order. In the first phase, two disks were collected at three sample collection times. One disk was used for viable bacterial cell counts, and the other disk was used for DNA extraction for real-time PCR and 16S rRNA sequence analysis. In the second phase, two disks were collected at each sample collection time for use in confocal laser scanning microscopy observations.

### Viable bacterial cell counts

Biofilm samples were sonicated for 5 min in sterile distilled water. The resulting bacterial suspension was diluted and spread onto Colombia blood agar plates (Becton, Dickinson and Company, Franklin Lakes, NJ, USA) and incubated in aerobic conditions for 24 h or anaerobic conditions using Anaero Pack Kenki (Mitsubishi Gas Chemical Company, Inc. Tokyo, Japan) for 48 h. Three plates were used for each concentration and each condition. The numbers of biofilm-forming bacteria were calculated by counting colonies.

### Quantification of total biofilm-forming bacteria by real-time PCR

Bacterial DNA was extracted with a DNeasy PowerSoil Kit (Qiagen, Hilden, Germany). Real-time PCR was performed in a total volume of 20 µl, composed of 10 µl of Power SYBR Green Master Mix (Applied Biosystems, Foster City, CA, USA), 1 µl of DNA, and the following universal bacterial 16S rRNA primers: 27F (AGRGTTTGATCMTGGCTCAG^39,40^) and 338R (TGCTGCCTCCCGTAGGAGT^41^). The final concentration of forward and reverse primers was 900 nM. The Applied Biosystems 7500 fast real-time PCR system (Thermo Fisher Scientific, Foster City, CA, USA) was used to estimate the numbers of biofilm-forming bacteria via a calibration curve method. The calibration curve was prepared using *Streptococcus mutans* ATCC 25175 genomic DNA.

### 16S rRNA gene sequencing

The V1–V2 region of 16S rRNA was amplified by using the primer set 27F and 338R. The Illumina library was prepared with the tailed PCR method, in accordance with the instructions of the “Illumina 16S Metagenomic Sequencing Library Preparation Guide”. Sequencing was performed with a MiSeq instrument (Illumina Inc.). The sequences were processed and clustered into operative taxonomic units (OTUs) with a 97% similarity cutoff using the Green Gene database. The results of sequences were analyzed using the Quantitative Insights Into Microbial Ecology (QIIME) pipeline.

### Confocal laser scanning microscopy observations

A modified biofilm staining method was used as previously described^42^. To analyze the structure of the biofilms, bacterial cells were stained with 4ʹ,6-diamidino-2-phenylindole (DAPI; Thermo Fisher Scientific), and EPS was stained with fluorescein isothiocyanate (FITC)-labeled concanavalin (Con A; Thermo Fisher Scientific) and FITC-wheat germ agglutinin (WGA; Thermo Fisher Scientific). The final concentrations of fluorescent labeling reagents were 125 ng/ml (FITC-ConA and FITC-WGA) and 105 ng/ml (DAPI). Biofilm samples on HA disks were immersed in aliquots of fluorescent labeling reagent for 30 min. A confocal laser scanning microscope (LSM 700, Carl Zeiss, Oberkochen, Germany) was used to observe the biofilms, and Imaris software (Imaris 5. 0. 1, Bitplane AG, Zürich, Switzerland) was used for image analysis.

### Statistical analysis

Friedman’s test was used to compare the numbers of living bacteria and total bacteria, as well as the volumes of total bacteria and EPS. The Wilcoxon signed rank test was used to compare the relative abundances of each genus. Differences among schedules with a *p*-value of < 0.05 were considered statistically significant. Statistical analyses and graphical outputs were performed with IBM SPSS Statistics (version 22.0, IBM SPSS Inc., Armonk, NY, USA).

## Results

### Numbers of biofilm-forming bacteria

**Figure 3:**
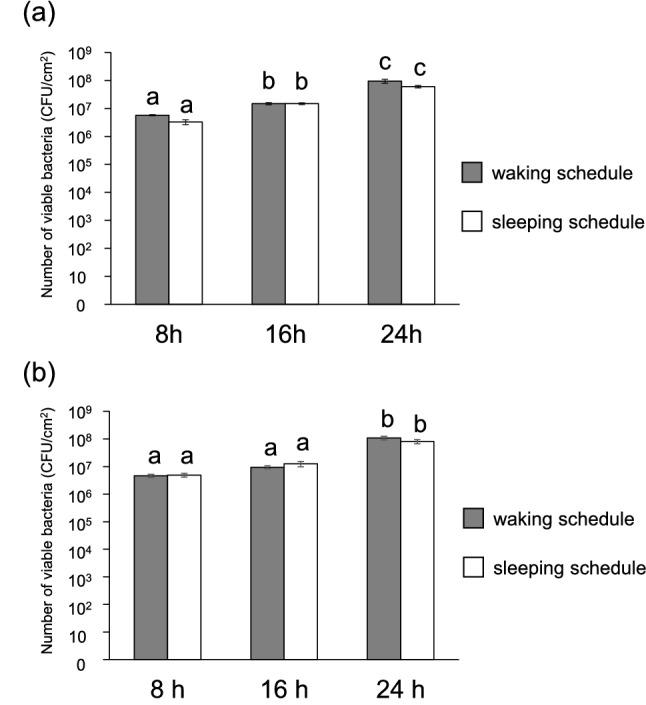
Numbers of viable biofilm-forming bacteria. (**a**,**b**) Numbers of viable biofilm-forming bacteria over time in dental biofilms from the waking and sleeping schedules, grown under aerobic (**a**) or anaerobic (**b**) conditions, as measured by counting colonies. Significant differences are represented by different letters (Friedman’s test at *p* < 0.05).

**Figure 4:**
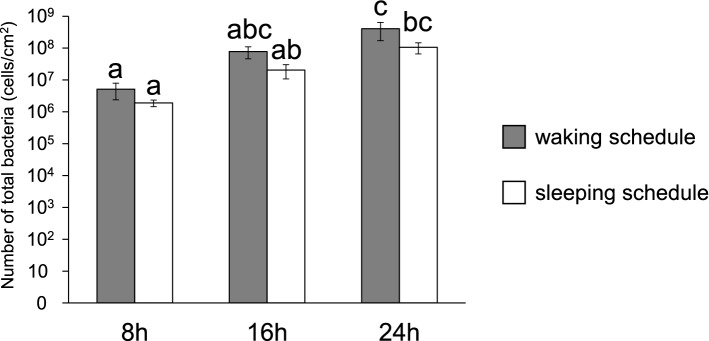
Numbers of total biofilm-forming bacteria. Numbers of total biofilm-forming bacteria over time in dental biofilms from the waking and sleeping schedules, as measured by real-time PCR. Significant differences are represented by different letters (Friedman’s test at *p* < 0.05).

Viable cell counts under aerobic and anaerobic conditions increased over time in both the waking and sleeping schedules (Fig. 3). Statistically significant increases were observed from 8 to 16 h, as well as from 16 to 24 h, under aerobic conditions in both the waking and sleeping schedules (Fig. 3a). The population of viable cells increased significantly from 16 to 24 h under anaerobic conditions (Fig. 3b). Similarly, a real-time PCR analysis revealed that the numbers of total bacteria increased over time in both schedules (Fig. 4). However, no significant differences were observed between the waking and sleeping schedules at each sample collection time (8 h, 16 h, 24 h) in terms of the viable cell count and real-time PCR analysis (Friedman’s test, *p* > 0.05).

### 16S rRNA gene sequencing

**Figure 5:**
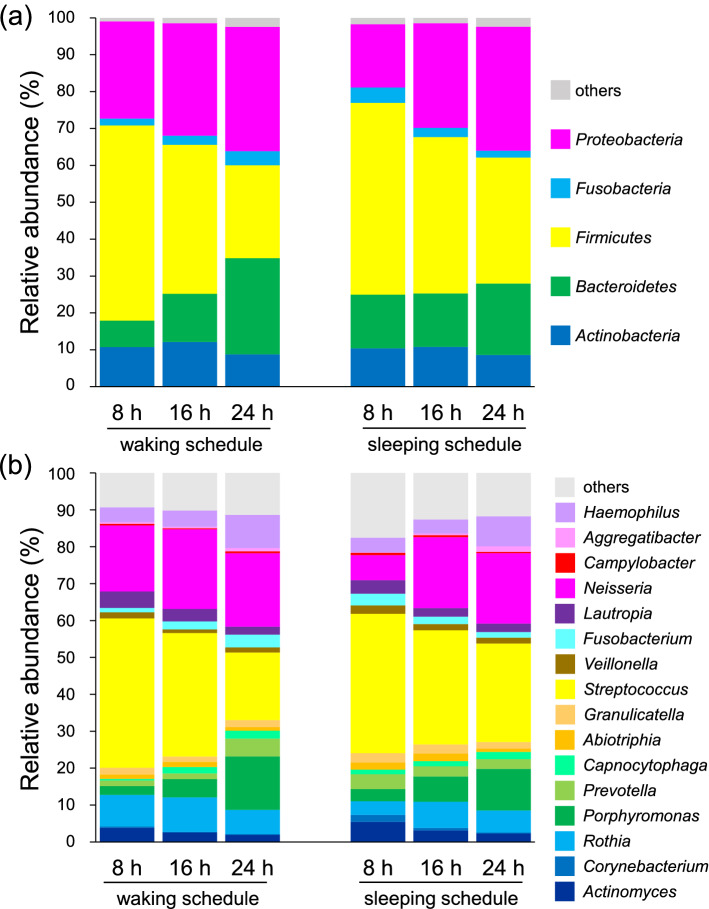
Relative abundance of bacterial taxa in dental biofilm. (**a**,**b**) The relative abundance at the phyla (**a**) and genus (**b**) levels of bacteria in dental biofilms grown during the waking or sleeping schedules. The bar colors indicate the taxa.

**Figure 6:**
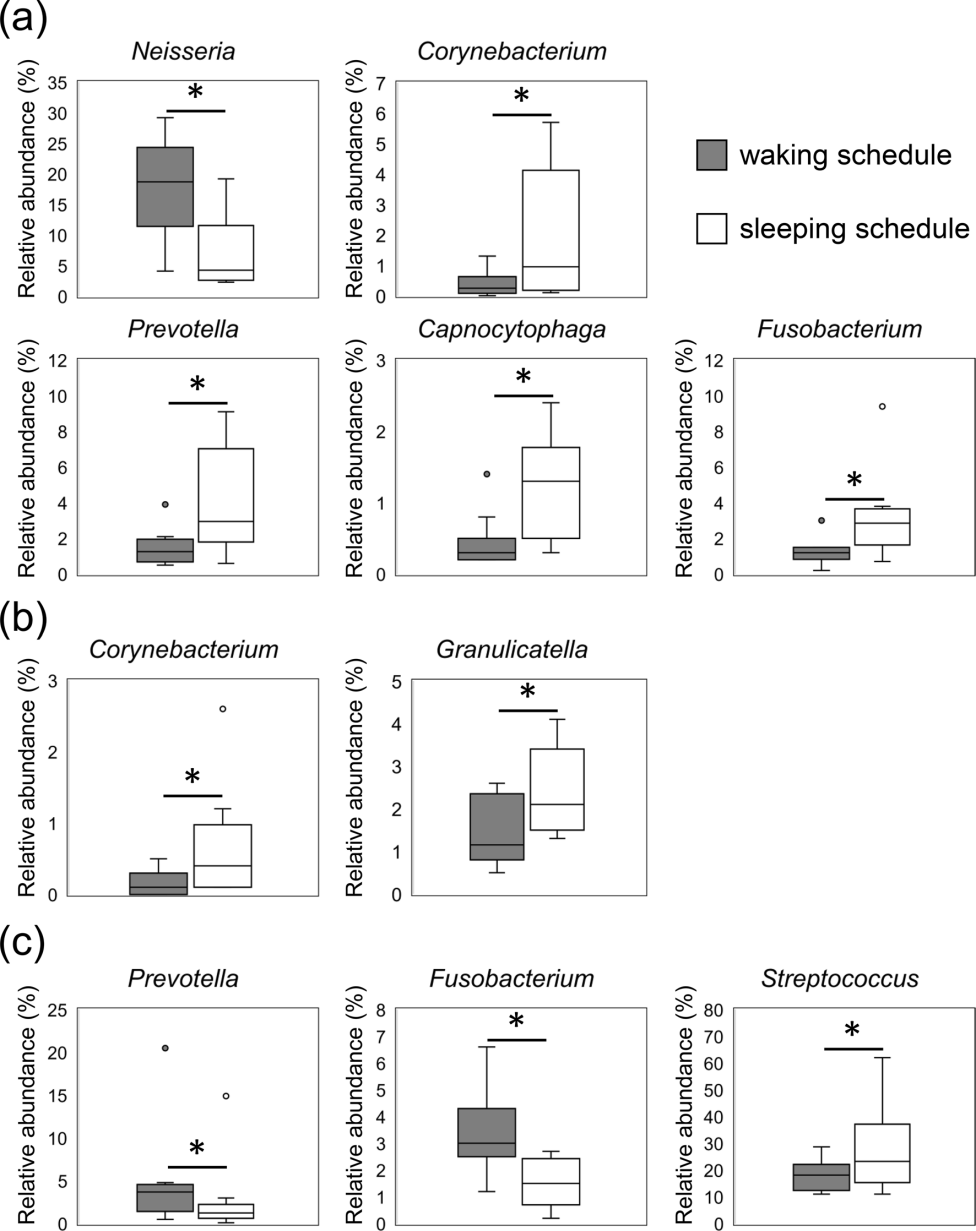
Genera with significantly different relative abundances between the waking and sleeping schedules. (**a**–**c**) Genera for which significant differences in abundance were observed between dental biofilms from the waking and sleeping schedules at 8 h (**a**), 16 h (**b**), or 24 h (**c**) (Wilcoxon signed rank test, *p* < 0.05). Circles represent outliers.

The microbial composition according to bacterial taxa is shown in Fig. 5. There were individual differences in the composition of bacterial taxa, but common tendencies were observed. Biofilm-forming bacteria on the hydroxyapatite (HA) disks mostly belonged to one of the following five phyla: Actinobacteria, Bacteroidetes, Firmicutes, Fusobacteria, and Proteobacteria (Fig. 5a). The relative abundance of Bacteroidetes was higher in the sleeping schedule than in the waking schedule at 8 h. Moreover, the relative abundance of Bacteroidetes increased over time in the waking schedule (7.2% of biofilm-forming bacteria at 8 h, 13.1% at 16 h, and 26.1% at 24 h), whereas there was almost no change over time in the sleeping schedule. The relative abundance of Firmicutes decreased over time in both the waking and sleeping schedules. The relative abundances at the genus level are shown in Fig. 5b. The relative abundance of *Neisseria* was lowest at 8 h in the sleeping schedule. The relative abundance of *Streptococcus* decreased over time in both schedules, especially from 16 to 24 h in the waking schedule. Genera with significant differences in relative abundance between the two schedules at each sample collection time are shown in Fig. 6. At 8 h, the relative abundance of *Neisseria* was significantly higher in the waking schedule than in the sleeping schedule; in contrast, the relative abundances of *Corynebacterium*, *Prevotella*, *Capnocytophaga*, and *Fusobacterium* were higher in the sleeping schedule (Wilcoxon signed rank test, *p* < 0.05). At 16 h, the relative abundances of *Corynebacterium* and *Granulicatella* were higher in the sleeping schedule than in the waking schedule. In contrast with the result at 8 h, the relative abundances of *Prevotella* and *Fusobacterium* at 24 h were significantly higher in the waking schedule than in the sleeping schedule (Wilcoxon signed rank test, *p* < 0.05). Biofilm samples collected upon awakening showed high abundances of obligate anaerobes, such as members of the *Prevotella* and *Fusobacterium* genera.

### Confocal laser scanning microscopy observations

**Figure 7:**
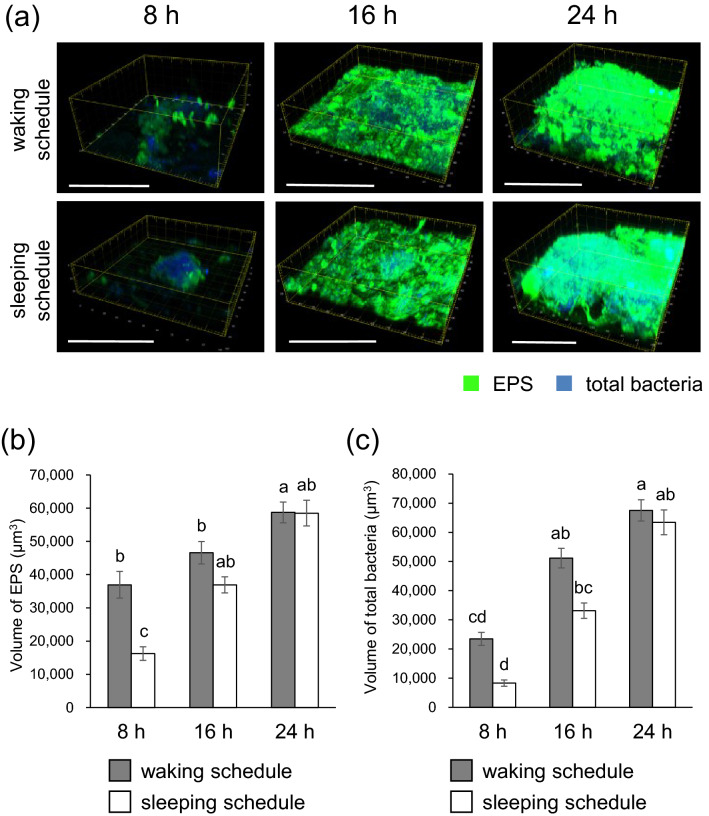
Confocal laser scanning microscopy images of in situ dental biofilm and the volumes of components in EPS-total bacteria staining. (**a**) Exopolysaccharide (EPS) labeled with FITC-ConA and FITC-WGA is depicted in green, and total bacteria labeled with DAPI are depicted in blue. (**b**,**c**) The volumes of EPS (**b**) and total bacteria (**c**). Significant differences are represented by different letters (Friedman’s test, *p* < 0.05).

Representative images of experimental biofilms on the HA disks are shown in Fig. 7a. In EPS–bacteria staining, blue-stained bacteria were observed surrounded by green-stained EPS (Fig. 7a). Small numbers of microcolonies were observed at 8 h in both schedules; however, the microcolonies in the sleeping schedule tended to be small and sparse (Fig. 7a). The numbers of EPS-enmeshed microcolonies increased over time and the biofilms covered nearly the entire HA disk surface at 16 h (Fig. 7a). The volume of EPS did not change from 8 to 16 h in the waking schedule (Fig. 7b), whereas the volumes of total bacteria gradually increased in both the waking and sleeping schedules (Fig. 7c). The volume of EPS was significantly greater at 8 h in the waking schedule than in the sleeping schedule (Fig. 5b, Friedman test, *p* < 0.05); it increased more rapidly from 8 to 16 h, as well as from 16 to 24 h, in the sleeping schedule than in the waking schedule.

## Discussion

The objective of this study was to investigate whether, and if so, how the characteristics of in situ dental biofilm are influenced by sleeping. Understanding changes in such characteristics may lead to the development of methods to effectively control dental biofilm, thereby preventing biofilm-associated oral diseases such as dental caries and periodontitis. The results obtained will have important implications for planning optimal oral hygiene, especially in providing scientific evidence for establishing effective timing for oral care. Because people typically brush their teeth every day, this study investigated the change in in situ biofilm characteristics on a daily basis. The applied study design has an important advantage for investigating the influence of sleeping. To research the impacts of sleeping, sample collection before and after sleeping would seem to be a reasonable method; however, a biofilm collected after sleeping would typically be older than a biofilm collected before sleeping, thus precluding an accurate investigation of the impact of sleeping. Here, we employed two schedules so that biofilms of the same age could be obtained, thus allowing the specific effects of sleeping to be investigated.

Viable cell counts and real-time PCR analyses revealed that the numbers of biofilm-forming bacteria increased over time under both the waking and sleeping schedules. We previously reported that the population of biofilm-forming bacteria increased in two steps: from the onset of biofilm formation to 48 h, and from 48 h onwards^37^. The findings of the present study are consistent with the findings of our previous study, in that the bacterial number increased over time. The number of bacteria in saliva was previously reported to increase rapidly during the night, and it was higher upon awakening in the morning^26^. The number of biofilm-forming bacteria was expected to increase along with an increase of the bacteria in saliva. Notably, no significant differences were observed in the populations of biofilm-forming bacteria between the waking and sleeping schedules at each sample collection time; this result suggests that the number of biofilm-forming bacteria is not affected by sleeping, although methodological limitations remain. The number of dental biofilm-forming bacteria is not closely related to the number of bacteria in saliva, although saliva is always present in the oral cavity, and the tooth surface is continuously in contact with saliva. The main reason for the increase in bacterial number within saliva during sleep is thought to be the decrease in salivary flow during this period. Furthermore, a decrease in salivary flow could reduce the level of salivary antibacterial agents, thus leading to bacterial growth in saliva. However, because bacteria within a biofilm are resistant to antibacterial agents, the population of biofilm-forming bacteria may be less affected than the planktonic cells in saliva by a reduction in the level of salivary antibacterial agents. The volumes of living bacteria (Supplemental data) and total bacteria obtained by CLSM observation correspond to the viable cell count and real-time PCR results, in that both the volume and the number of bacteria increased over time.

In the present study, changes in bacterial composition after sleep were observed via a 16S rRNA sequence analysis. The bacteria contained in the in situ dental biofilm belonged to one of the following five phyla: Actinobacteria, Bacteroidetes, Firmicutes, Fusobacteria, and Proteobacteria. These results are consistent with those of some previous reports^5,6^. At the genus level, the relative abundances of the genera *Prevotella* and *Fusobacterium* increased in 8-h- and 24-h-old biofilms after sleeping. A previous study revealed that the abundances of *Porphyromonas*, *Prevotella*, and *Fusobacterium* increased rapidly at 48 h after the onset of formation of an in situ dental biofilm^37^. In the report by Socransky et al.^43^, *Fusobacterium nucleatum*, *Prevotella intermedia*, and *Prevotella nigrescens* were included in the orange complex, which was closely associated with periodontitis. The abundance of *Porphyromonas* increased over time under both the waking and sleeping schedules, and no significant difference was observed between these two schedules at any sample collection timepoint. In a previous study, we reported that the relative abundance of *Porphyromonas* also increased at 24 h after biofilm formation, compared with that at 8 h post-biofilm formation, with a less remarkable change as observed after 48 h post-biofilm formation^37^. Thus, the increase in the abundance of *Porphyromonas* observed here may be simply related to the process of biofilm maturation; unlike *Fusobacterium* and *Prevotella*, for *Porphyromonas*, abundance may not be affected by sleep.

Saliva plays an important role in the host defense system^44^. Saliva contains various antibacterial agents, such as mucins, lysozyme, lactoferrin, statherin, cystatins, histatins, and immunoglobulins (especially sIgA). Salivary flow and swallowing contribute to the removal of bacteria and are important for balancing the oral microbiome. The environment of the oral cavity, including the saliva components, oxygen concentration, and salivary flow, is presumed to affect the dental biofilm microbiota.

The relative abundance of *Neisseria* was significantly higher in the waking schedule than in the sleeping schedule at the 8 h timepoint. Bacteria belonging to the genus *Neisseria* are considered indigenous bacteria. *Neisseria* reportedly comprises 10% of dental plaque from 1 to 3 days, but its population decreases after 5 days^45^. In the present study, the population of *Neisseria* was lower in 8-h-old dental biofilm formed during sleep than in 8-h-old dental biofilm formed during the daytime; it increased rapidly from 8 to 16 h during the sleeping schedule. In contrast, during the waking schedule, its abundance was nearly constant from 8 to 24 h. The abundance of *Neisseria* is presumably dependent on the environment in the early stages of biofilm formation but is not affected 8 h after the start of biofilm formation.

In the present study, the relative abundance of *Corynebacterium* remained higher in the sleeping schedule than in the waking schedule from 8 to 24 h. *Corynebacterium matruchotii* is considered important for the mineralization of dental biofilm and the formation of dental calculus^46^. In addition to being a risk factor related to periodontitis, dental calculus encourages the accumulation of oral bacteria on dental surfaces. The relative abundance of *Corynebacterium* is reportedly 1% in 3-day-old dental plaque, and this abundance increases after 3 days^45^. Therefore, in situ dental biofilm that begins to form at night maintains a high abundance of *Corynebacterium* and may be closely associated with periodontal disease.

The volume of EPS was significantly greater for the waking schedule than for the sleeping schedule at 8 h. The microbial composition of 8-h-old biofilm differed between the sleeping and waking schedules. As previously reported, saliva is the main source of nutrition for dental biofilm during sleep^47^. Notably, the composition of saliva during sleep differs from its composition during wakefulness^25,48^; for example, the amounts of sugar and proteins in saliva are lower during sleep. This is potentially because sleeping time serves as a “famine period”^49^. Some proteins in saliva assist bacterial cells in adhering to the tooth surface as a pellicle^50^. During sleep, the lower concentrations of proteins and different pellicle characteristics may affect the structure of the resulting biofilm. Therefore, changes in the bacterial composition of in situ dental biofilm and the oral environment during sleep can result in reduced EPS production by biofilm-forming bacteria.

There have been multiple investigations of the ideal timing and frequency of oral care (including toothbrushing). Lang et al.^51^ reported that oral hygiene procedures at 48-h intervals could prevent gingivitis in healthy volunteers. However, for caries prevention, toothbrushing is recommended after every meal because the main cause of dental caries is the acidic environment created by dental bacteria^52,53^; notably, the enamel surface pH significantly decreases after exposure to sucrose^54^.

The results of the present study suggest that the number of biofilm-forming bacteria is not affected by sleep, whereas the relative abundances of obligates anaerobes (e.g., *Fusobacterium* and *Prevotella*) are higher after waking than during the daytime. Importantly, obligate anaerobes in dental biofilm are associated with periodontitis^30^. Therefore, effective periodontitis prevention may involve the removal of dental biofilm that contains more obligate anaerobes upon awakening. However, the pathogenic characteristics of obligate anaerobes in dental biofilm were not investigated in this study, so further analysis is required.

There are some limitations in this study. First, this research focused on only healthy subjects without dental caries or periodontitis. For establishing methods of controlling such oral diseases in individuals suffering from them, additional investigations into the characteristics of dental biofilm in patients with oral diseases are needed. Second, this study used 16S rRNA sequencing to examine the bacterial composition in dental biofilm at the genus level. To better clarify the differences in biofilm properties between periods of sleep and wakefulness, future work will need to evaluate function, especially pathogenic properties or metabolic activity, and conduct a species-level analysis. Third, sleep was self-reported and was not monitored because it is difficult to accurately monitor sleeping. The biofilm composition may be different in individuals with sleep disorders, such as insomnia and sleep apnea, or who breath through their mouths rather than their noses during sleep; if these issues can be clarified by sleep monitoring, it could allow for oral care to be tailored to individual patients.

In conclusion, dental biofilm containing obligate anaerobes was formed, and the dental biofilm structure changed during sleep, whereas the numbers of biofilm-forming bacteria did not change during sleep. The findings of this study will aid in the establishment of evidence-based methods for improved oral care.

## Supplementary Information

Supplementary Figure S1.

## Data Availability

The datasets used and analyzed during the current study are available from the corresponding author on reasonable request.
