# Supplementary material for: Impacts of sleep on the characteristics of dental biofilm
Source: Sci Rep. 2021 Jan 8;11:138. doi: 10.1038/s41598-020-80541-5 (PMC7794455; doi:10.1038/s41598-020-80541-5)
Supplement: Supplementary file 1 — Supplementary Figure S1. [file 41598_2020_80541_MOESM1_ESM.pdf]

Impacts of Sleep on the Characteristics of Dental Biofilm

Maki Sotozono, Nanako Kuriki, Yoko Asahi, Yuichiro Noiri, Mikako Hayashi, Daisuke Motooka, Shota Nakamura, Hiroyuki Machi, Tetsuya Iida and Shigeyuki Ebisu

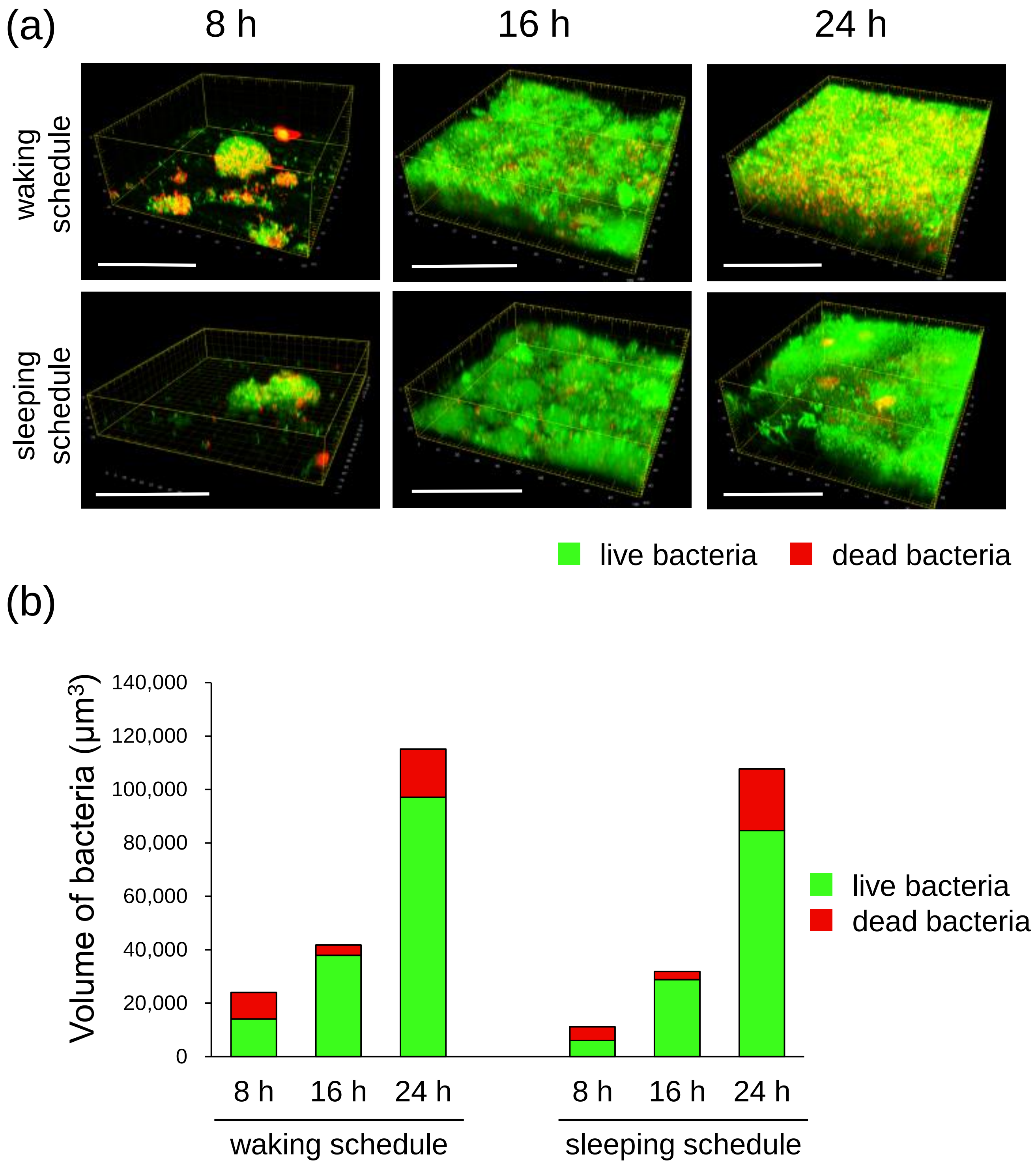

Supplementary Fig S1. The Confocal laser scanning microscopy images of in situ dental biofilm and the volumes of components in LIVE/DEAD® staining

(a) Biofilms on the HA disks were stained using a LIVE/DEAD® BacLight Bacterial Viability Kit (Thermo Fisher Scientific) for 30 min. The live cells are depicted in green, and the dead cells in red. (b) The volumes of both live bacteria and total bacteria increased over time, results that agree with the findings from a previous report (npj Biofilms Microbiomes 2016; 10: 2:16018). There were no significant differences in the volumes of live and total bacteria between the waking schedule and sleeping schedule at any sampling timepoint. The percentage of dead bacteria was significantly higher at 8 h than at 16 h or 24 h for both experimental schedules; however, no significant difference in this percentage was observed between biofilms from the waking schedule and sleeping schedule at any sample collection timepoint.
